# Deep brain stimulation induced effects in a network of ventral intermediate neurons

**DOI:** 10.1186/1471-2202-14-S1-P427

**Published:** 2013-07-08

**Authors:** Caroline T Golden, Dipankar Nandi, Peter Bain, Nada Yousif

**Affiliations:** 1 Department of Bioengineering, Imperial College London, London, SW7 2AZ, UK; 2 Division of brain sciences, Imperial College London, London, W86 8RF, UK

## 

**Figure 1:**
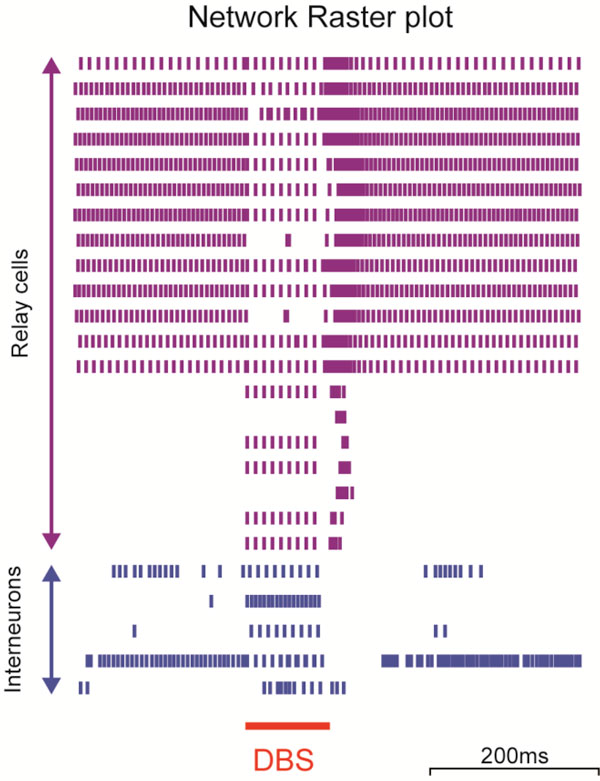
**Raster plot of the modelled Vim nucleus network behaviour for a therapeutic DBS parameter setting (frequency = 100 Hz, pulse width = 60µs and amplitude = 2V)**. The 20 relay cells are shown with the five interneurons. The red line indicates the time during which DBS was simulated as being on (100 ms).

Essential tremor (ET) is a common movement disorder successfully treated by deep brain stimulation (DBS) [1], a surgical therapy involving the implantation of electrodes into disorder specific nuclei in the brain through which high frequency electrical pulses are chronically administered. While the therapy is clinically beneficial, the mechanisms at the level of the changes occurring in the surrounding tissue are unknown [2]. If we better understood these mechanisms, we could optimise the parameters setting process and minimise unwanted side effects in patients. To investigate these mechanisms we model a small network of excitatory relay cells and inhibitory interneurons within the ventral intermediate (Vim) nucleus of the thalamus. The network model was constructed using previously published multicompartment models [3] in NEURON 7.0 and was set up to reflect the known connectivity of the Vim nucleus. The DBS induced electric field was simulated in a finite element environment using COMSOL Multyphysics 3.3 by solving the Laplace equation within a 3-dimensional geometric model of the DBS electrode within a homogenous cylinder of brain tissue. The findings show that a variety of neural activity patterns are induced by therapeutic and non-therapeutic parameters derived from empirical and clinical reports. The network in isolation displayed tonic firing patterns in most of the relay cells, with strong inhibition in the remaining excitatory cells. The prevalence of interneuron-relay synaptic connections may be influential in this respect by acting as a gain control mechanism for relay cells, a concept which has been alluded to in prior investigations [4]. Once DBS was applied (Figure 1A), the network behaviour changed considerably. A combined effect of excitation and inhibition, which was dependent on spatial positioning within the stimulus field, decreased the overall activity of the network and increased the prevalence of hyperpolarisation-induced burst firing at DBS offset (Figure 1A). The simulations revealed this to be one of the most discernible network-level changes between therapeutic and non-therapeutic DBS simulations, which may relate to the links made in the literature between burst firing and tremor oscillations [5]. This is the first time that the effect of the DBS induced electric field on a thalamic network has been investigated and leads to a clearer understanding of the neuronal changes in the VIM nucleus.
